# Bio-Inspired nacre-like nanolignocellulose-poly (vinyl alcohol)-TiO_2_ composite with superior mechanical and photocatalytic properties

**DOI:** 10.1038/s41598-017-02082-8

**Published:** 2017-05-12

**Authors:** Yipeng Chen, Hanwei Wang, Baokang Dang, Ye Xiong, Qiufang Yao, Chao Wang, Qingfeng Sun, Chunde Jin

**Affiliations:** 1 School of Engineering, Zhejiang A & F University, Hangzhou, Zhejiang Province, 311300 P.R. China; 2 Key Laboratory of Wood Science and Technology, Zhejiang Province, 311300 P.R. China

## Abstract

Nacre, the gold standard for biomimicry, provides an excellent example and guideline for assembling high-performance composites. Inspired by the layered structure and extraordinary strength and toughness of natural nacre, nacre-like nanolignocellulose/poly (vinyl alcohol)/TiO_2_ composites possessed the similar layered structure of natural nacre were constructed through hot-pressing process. Poly (vinyl alcohol) and TiO_2_ nanoparticles have been used as nanofillers to improve the mechanical performance and synchronously endow the superior photocatalytic activity of the composites. This research would be provided a promising candidate for the photooxidation of volatile organic compounds also combined with outstanding mechanical property.

## Introduction

As performance limitations of conventional structural materials, one of the major scientific challenges for the 21st century is the development of new multifunctional and high performance materials to support advances in diverse strategic fields, ranging from building and transportation to biotechnology and energy^1^. In the process of evolution, nature has found masterly ways to produce lightweight, strong, and high-performance materials with exceptional properties and functionalities^2^. As history advanced, some materials such as bone, wood and shells were slowly replaced by synthetic compounds that offered improved performance.

The most studied model among these biological materials is the nacreous part in some mollusk shells that consists of about 95 wt.% of brittle aragonitic CaCO_3_ and 5 wt.% of organic materials^3^. Nacre is twice as strong as and 1000-fold tougher than its constituents. From a mechanical point of view, nacre is often simplified as a binary composite, in which hard, two-dimensional (2D) aragonite platelets and soft biopolymer layers are alternately stacked into a brick-and-mortar structure^4, 5^. Thus, mimicking the brick-and-mortar architecture of nacre by assembling different types of 2D platelets and polymer matrices is a viable approach for designing new materials. For nacre, the strategies used for producing its artificial counterparts can be categorized into three groups: the layer-by-layer technique^6^, the self-assembly technique^7^ and the slurry-based freeze-casting^8^/magnetic-field-assisted slip-casting^9^ and sintering technique^10^. To the best of our knowledge, no reports about the production of nacre-like NLC/inorganic nanoparticles composite by hot-pressing process.

Because of its abundance and sustainability, plant cellulose and cellulosic nanomaterials have attracted growing interest as an alternative to synthetic materials, especially as a filler and reinforcement for composites^11^. Lignocellulose is the most abundant, low cost, biodegradable, and environmentally friendly biopolymer with a hierarchical structure^12^. That mechanical fibrillation can provide sufficient external friction to rapidly destroy the cell wall of the lignocellulose, and obtain uniform nanolignocellulose (NLC). The NLC produced through mechanical fibrillation using a colloid mill mince master^13^. Recently, metal and metal oxide nanostructures have been used as fillers in high-end applications such as photocatalyst, antibacterial, supercapacitor, and magnetic applications. Among others, titania (TiO_2_) is considered to be one of the economical and environmentally friendly catalysts and demonstrates high oxidizing power on exposure to UV light^14^. Therefore, along with the increasing research interest in organic–inorganic nanocomposites often presenting the best properties of each of the components in a synergic way, dispersing nanoparticle ferrites (like TiO_2_) in NLC matrixes provides a feasible pathway for producing photocatalytic multifunctional nanocomposites.

Herein, inspired by the layered aragoniteplatelet/nanofibrillar chitin/protein structure of nacre, nacre-like composites based on nanolignocellulose(NLC)/poly (vinyl alcohol) (PVA)/titania (TiO_2_) through hot-pressing process was constructed^15, 16^. Through the mechanical fibrillation and hot-pressing process, TiO_2_ dispersed in NLC matrixes to produce photocatalytic properties nanocomposites. The nacre-like composites achieves an excellent mechanical property, superior to other layered cellulose/polymer binary composites. Combined without standing mechanical, their photocatalytic properties make these composites promising candidates in the photooxidation of volatile organic compounds(VOCs) and other organic pollutants applications.

## Results

**Figure 1:**
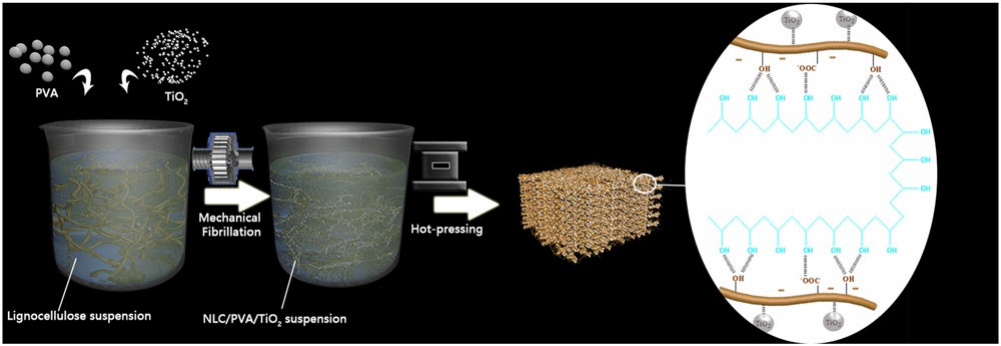
Schematic illustration of fabrication of Nacre-like NLC/PVA/TiO_2_ composite

The hybrid layered structure model of resultant nacre-like composite was proposed as shown in Fig. 1. Firstly, the lignocellulose suspension mixed with 1 wt.% TiO_2_ and 4 wt.% PVA was added into SuperMassColloider at 1,500 rpm. Subsequently, lignocellulose/PVA/TiO_2_ suspension was fed into the disk grinder continuously for 6 hours through a loop consisting of a peristaltic pump and plastic tubing. Finally, after the redundant water of NLC/PVA/TiO_2_ suspension was filtered, the composites were hot-pressed at 200 °C, 2.5 MPa and cured into the layered board. The NLC platelets with TiO_2_ nanoparticles were adhered by PVA through strong hydrogen bonds.

**Figure 2:**
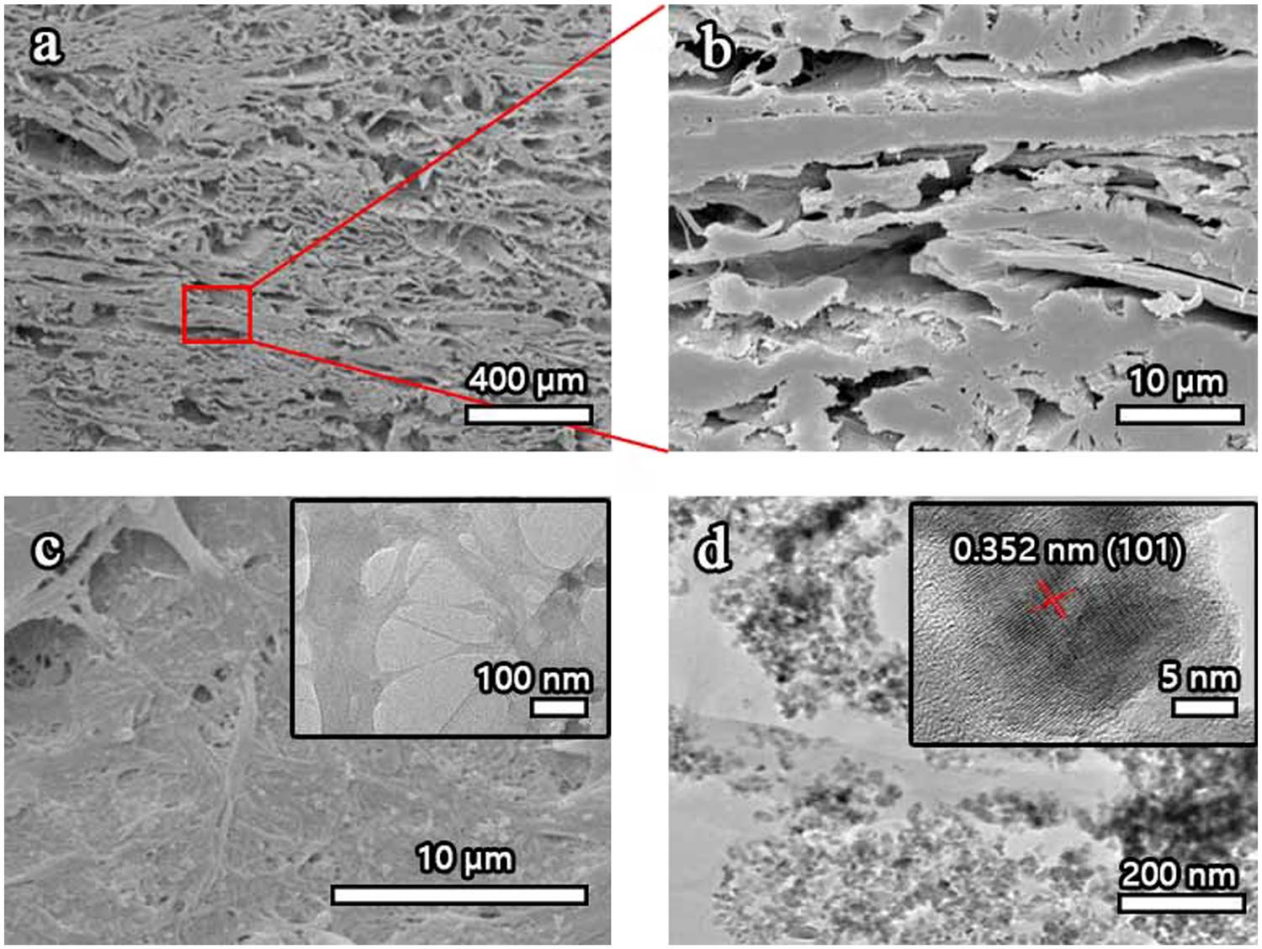
(**a**) Cross-sectional SEM image of NCL/PVA/TiO_2_ composite, showing its many layered structure. (**b**) Magnified image of one layer of (**a**). (**c**) Magnified top view of NCL/PVA/TiO_2_ composite, the inset is the HRTEM image of pure NLC. (**d**) TEM images for NCL/PVA/TiO_2_ composite, the inset is the HRTEM image of TiO_2_.

Scanning electron microscopy (SEM) revealed the multi-layered structure of the NCL/PVA/TiO_2_ composite. Figure 2a noted that each free standing layer inside the composite had a thickness of several hundred nanometers (Fig. 2b) and was composed of randomly entangled nanowires (Fig. 2c). As shown in the HRTEM image (inset of Fig. 2c), the pure NLC typically had diameters of 50–100 nm, these were stacked with each other through van der Waals force. Size of NLC/TiO_2_ containing of TiO_2_ were also shown in Fig. 2d, this confirmed the uniform distribution of the TiO_2_ in the NLC matrix, although particles seem to aggregate to some extent. It could be seen that the TiO_2_ exhibited spherical morphology. The HRTEM (inset of Fig. 2d) images and their size distributions showed that the mean diameters and standard deviation of TiO_2_ were about 9.93 ± 2.42 nm.

**Figure 3:**
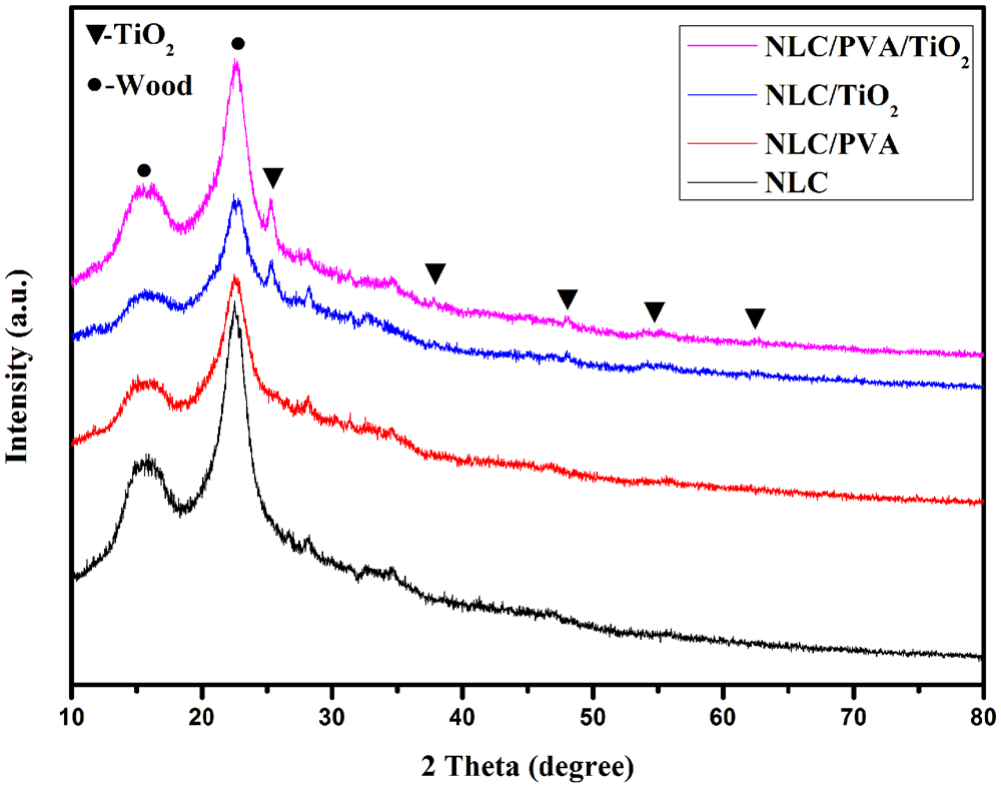
The XRD patterns for the NLC, NLC/PVA composite, NLC/TiO_2_ composites and NLC/PVA/TiO_2_ composite.

Figure 3 showed the XRD patterns for the NLC, NLC/PVA composite, NLC/TiO_2_ composites, NLC/PVA/TiO_2_ composite. The main peak at 2θ = 15.5° and 22.4°, which corresponded to the crystalline region of the cellulose in wood^17^. Besides, the other peaks at 2θ = 25.2°, 37.8°, 47.9°, 54.1°, 62.6° represented the crystal plane at (101), (103, 004 and 112), (200), (105 and 211), (204), respectively (anatase PDF 21–1272)^18^, were observed in the NLC/TiO_2_ composite and NLC/PVA/TiO_2_ composite. Additionally, there was no obvious absorption peak of PVA crystal in NLC/PVA and NLC/PVA/TiO_2_ composites showed the crystal structure of PVA damage in the grinding process^19^, this illustrated that PVA is fully converted to amorphous state.

The FTIR spectra of the NLC, NLC/PVA, NLC/TiO_2_ and NLC/PVA/TiO_2_ composites was shown Figure S1. The absorption bands at 3405 cm^−1^, 1650 cm^−1^ and 1420 cm^−1^ corresponded to the bands of the O-H bond, O-H bending vibration and C-H deformation, respectively. Most of the peaks represent major cell wall components in the nanolignocellulose such as cellulose (1161, 896 cm^−1^), hemicelluloses (1732, 1110, 1055 cm^−1^) and lignin (1599, 1507, 1231 cm^−1^). The spectrum clearly reveals the major peaks of NLC/PVA and NLC/PVA/TiO_2_ composites at 1090–1150 cm^−1^, 2850–3000 cm^−1^, and 3200–3570 cm^−1^ were due to C-O stretching, C-H broad alkyl stretching band, and hydrogen bonded of PVA, respectively. The characteristic peaks of NLC/TiO_2_ and NLC/PVA/TiO_2_ composites at 486 cm^−1^ and 628 cm^−1^ was attributed to the stretching vibration of Ti-O bond assigned to TiO_2_ nanoparticles. In addition, a strong interaction between the hydroxyl groups of nanolignocellulose, PVA and TiO_2_ through hydrogen bonds led to the absorption bands of the O-H bond of the NLC/PVA and NLC/TiO_2_ composites shift to 3417 and 3415 cm^−1^ compared with that of NLC (in 3427 cm^−1^). Thus, it was deduced that PVA molecules and TiO_2_ combined with the nanolignocellulose by O-H bonds.

**Figure 4:**
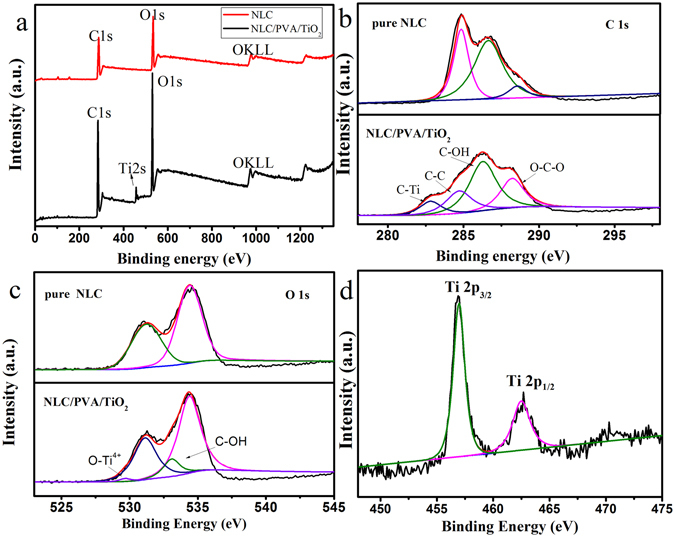
XPS spectra of (**a**) survey spectrum, (**b**) C1s, (**c**) O1s in pure NLC and NLC/PVA/TiO_2_ composite, respectively and (**d**) Ti 2p in NLC/PVA/TiO_2_ composite.

The XPS spectra of pure NLC and NLC/PVA/TiO_2_ composite were shown in Fig. 4. The wide scan spectra (Fig. 4a) of pure NLC, and NLC/PVA/TiO_2_ composite exhibited two major peaks with binding energy 285.6 and 530.3 eV corresponded to the C 1 s and O 1 s of cellulose, respectively^17^. However, in the NLC/PVA/TiO_2_ composite, an additional peak was observed at a binding energy of 458.9 eV corresponded to the Ti 2p of TiO_2_^14^. In order to further understand the structure, the high-resolution XPS spectra were examined. Figure 4b showed the C (ls) spectrum of the pure NLC, and NLC/PVA/TiO_2_ composite. The major peak at about 286.4 eV corresponded to C-O functional group which abounds in cellulose and C-OH functional group which abounds in PVA^20^. A well distinguished peak at 284.8 eV corresponded to the C-C bonds, while the third one which appears as a shoulder at about 287.9 eV corresponded to the O-C-O bond. In addition, there might be a very small peak situated near 282.8 eV which was attributable to the C-Ti bonds. In pure NCL (Fig. 4c), two peaks were observed in the high-resolution spectra of O 1 s at 531.0 and 533.4 eV. However, in the high-resolution spectra of the NCL/PVA/TiO_2_ composite the additional peak observed at 533.1 eV and 529.0 eV could be attributed to the C-OH bonds and the O-Ti^4+^, respectively^14^. In the Fig. 4d, two peaks were observed at 457.0 and 463.7 eV. The two peaks at 457.0 eV were due to Ti 2p3/2, and 463.7 eV was due to Ti 2p1/2^14^. This further confirmed the presence of TiO_2_ in the NLC/PVA/TiO_2_ composite.

**Figure 5:**
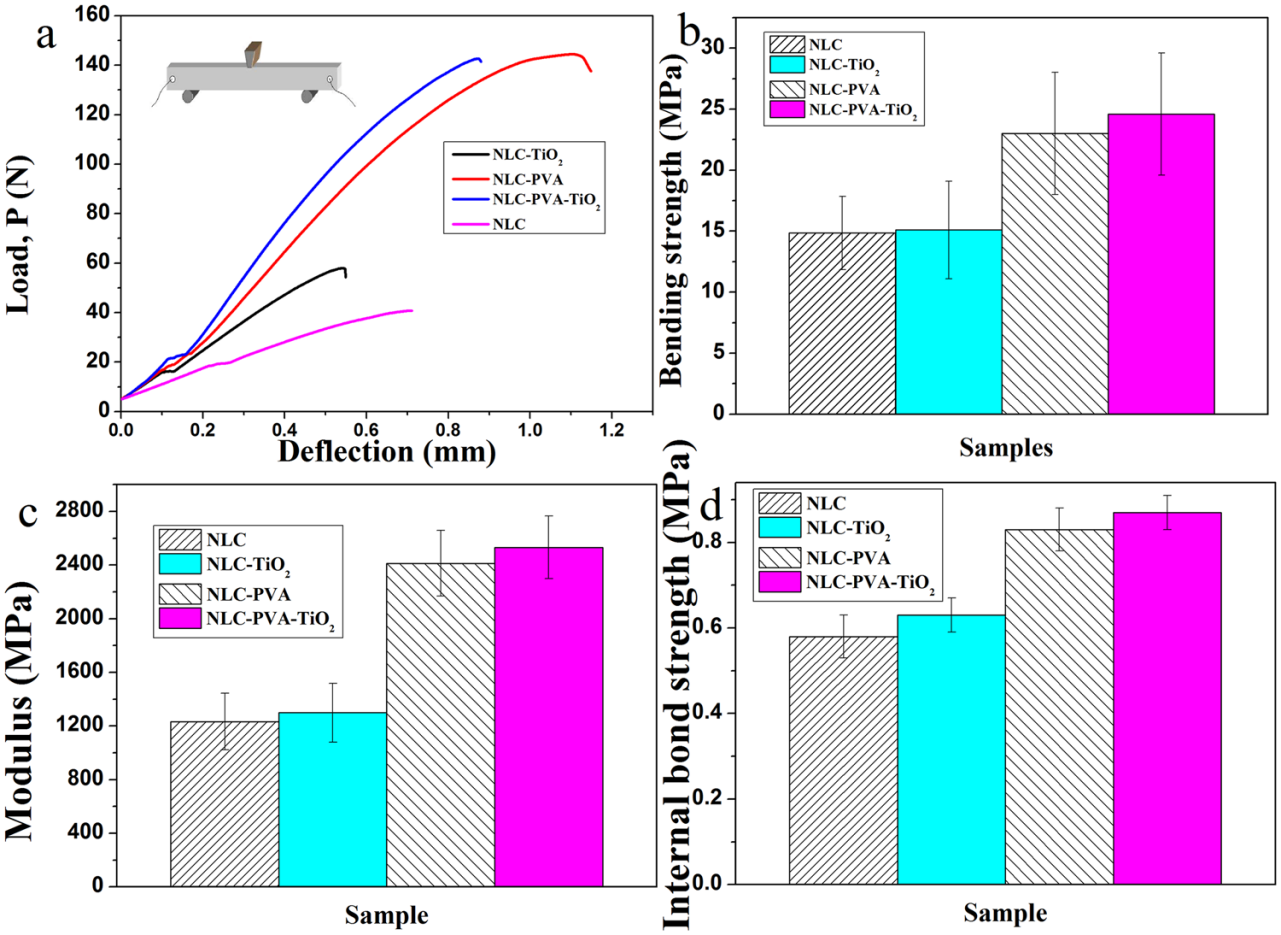
(**a**) The three-point bending tests force-displacement curves, (**b**) binding strength, (**c**) elasticity modulus and (**d**) internal bond strength of pure NLC, NLC/TiO_2_ composite, NLC/PVA composite and NLC/PVA/TiO_2_ composite.

The three-point bending tests force-displacement curves of the nacre-like composite were plotted in Fig. 5a. In this study, the NLC/PVA composite and NLC/PVA/TiO_2_ composite were indeed tougher than the pure NLC and NLC/TiO_2_ composites, respectively. The nacre-like composites showed excellent integration of binding strength and elasticity modulus, greater than other binary composites (pure NLC, NLC/TiO_2_ composites and NLC/PVA/TiO_2_ composite). The average binding strength (Fig. 5b), elasticity modulus (Fig. 5c) and internal bond strength (Fig. 5d) reached 24.6 ± 5 MPa, 1255.5 ± 41 MPa and 0.87 ± 0.04 MPa, respectively.

The contribution to the synergistic strength of nacre-like composites was the interconnectivity between two layers of NLC/TiO_2_composite through PVA. If PVA adhesive was absent, the NLC/TiO_2_composite layers tend to aggregate^5, 21^. The interaction between anionic two layers of NLC/TiO_2_ composite was relatively weak. In addition, NLC/TiO_2_ composite was quite rigid. Direct stress transfer between two rigid components was often deficient, as indicated by multiple recent studies^22, 23^. As a result, the pure NLC and NLC/TiO_2_ composites without PVA exhibited relatively weak mechanical properties with internal bond strength of 0.57 MPa and 0.63 MPa (binding strength of 14.8 MPa and 15.1 MPa). The contrast comparison clearly showed the importance of PVA adhesion between two layers of NLC/TiO_2_composite in the nacre-like composites.

**Figure 6:**
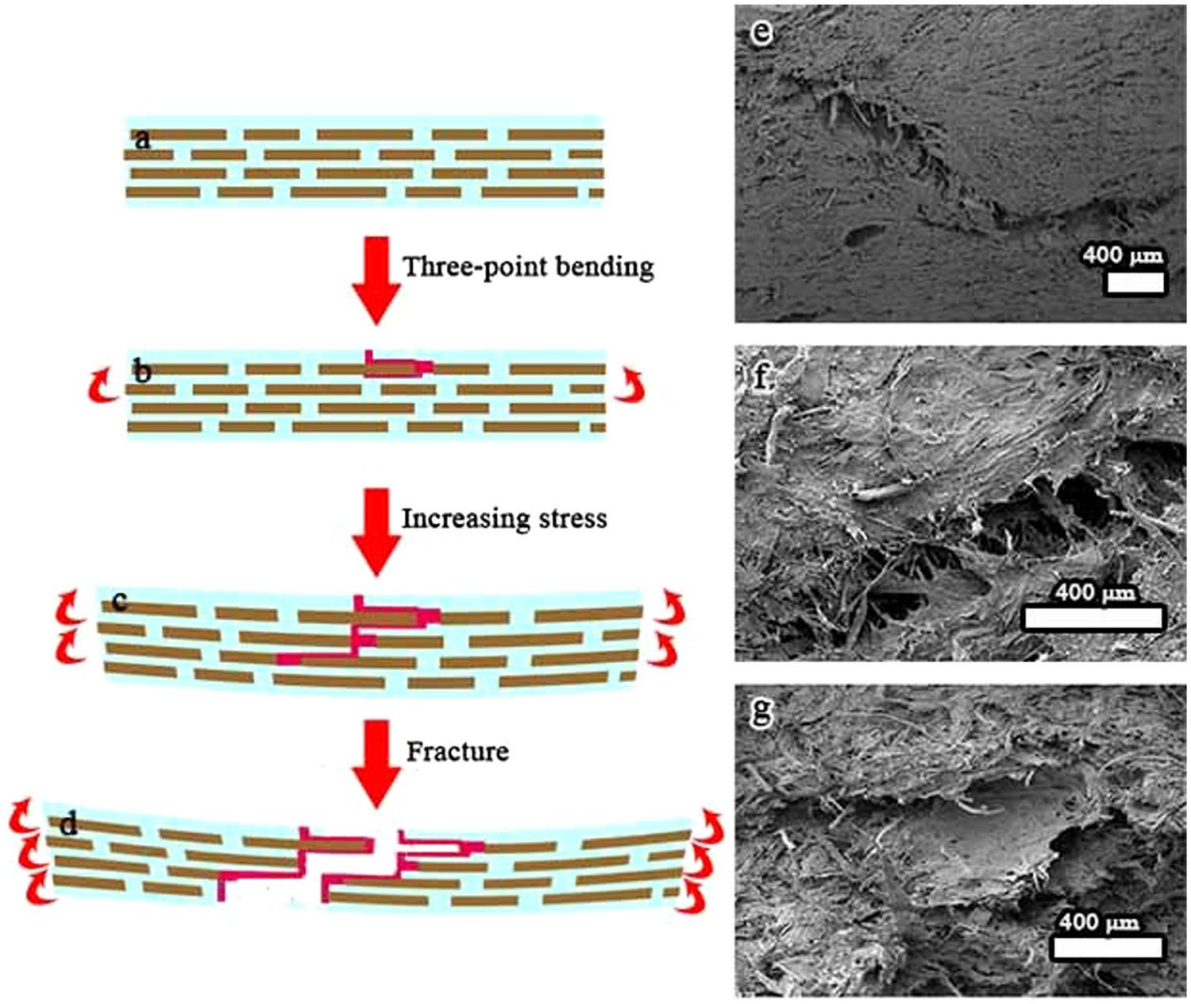
(**a**–**d**) Proposed synergistic mechanism of 2D NLC platelets. (**a**) Simplified structural schematics, showing the alternate arrangement of2D NLC platelets. (**b**) Under three-point bending, the NLC platelet starts to slide and deflect crack, (**c**) activates thepotential sliding of adjacent multiple NLC platelets. (**d**) The composite finally fails under NLC platelets facture mode. (**e**–**g**) Fracture morphology of the nacre-like composite, (**e**) crack deflection between layers and crack branching, (**f**) crack bridging, (**g**) NLC platelets’ fracture surface.

In terms of the fracture morphology of the nacre-like composites, a crack extension model was proposed to illustrate the synergistic toughening of NLC/PVA/TiO_2_ composite, as shown in Fig. 6a–d. First, a deflected crack induced by an NLC platelet encounters another NLC platelet (Fig. 6b). The bridging effect of platelets could generate obvious resistance to the sliding of adjacent NLC platelets (strain hardening). The enhanced stress was transferred to next layer of NLC platelets and activates the potential sliding of adjacent multiple NLC platelets (Fig. 6c). With crack extension, such crack deflection, bridging, and activation of multiple potential sliding sites are accumulative in a step fashion until the material fractures. In addition, the delamination process was retarded by the infiltrated PVA binding layers and thus further dissipates energy^24^.

To explore the mechanism of the toughness, fracture morphology of the nacre-like composites was carefully examined, as shown in Fig. 6e–g. The laminated nacre-like composite leads to crack branching, crack deflection and crack trapping (Fig. 6e). At the tip of microcracks, crack bridging, fibril pull-out and fracture were observed (Fig. 6f). The NLC platelets were pulled out and the NLC fractured in the process of three-point bending (Fig. 6g). The feature of the fracture surface indicated that NLC platelet sliding happened not only on the fracture surface but also in the interior of the NLC/PVA/TiO_2_ composite, which might be responsible for the improved fracture energy of the NLC/PVA/TiO_2_ composite. With crack extension, such crack deflection, bridging, and activation of multiple potential sliding sites were accumulative in a step fashion until the material fractures. This deformation process was also reflected by the strain hardening in the nonlinear force-displacement curve (Fig. 5a) and was akin to the deformation of hydrated nacre^25^.

The photocatalytic activity of NLC, NLC/PVA, NLC/TiO_2_ and NLC/PVA/TiO_2_composites was studied by degrading methyl orange in the presence of UV light for the different exposure times. The degradation of methyl orange (MO) by TiO_2_ in the presence of UV light and its mechanistic pathway have been well documented, as shown below^26^.

1 $$Ti{O}_{2}+hv\to Ti{O}_{2}({h}_{vB}^{+}+{e}_{CB}^{-})$$

2 $${h}_{vB}^{+}+{e}_{CB}^{-}\to {\rm{heat}}$$

3 $$Ti{O}_{2}({e}_{CB}^{-})+{O}_{2}\to Ti{O}_{2}+{O}_{2}^{\cdot -}$$

4 $${O}_{2}^{\cdot -}+{H}^{+}\to H{O}_{2}^{\cdot }$$

5 $$H{O}_{2}^{\cdot -}+{O}_{2}^{\cdot -}+{H}^{+}\to {H}_{2}{O}_{2}+{O}_{2}$$

6 $$2H{O}_{2}^{\cdot -}\to {H}_{2}{O}_{2}+{O}_{2}$$

7 $$H{O}_{2}^{\cdot -}+{H}^{+}+Ti{O}_{2}({e}_{CB}^{-})\to Ti{O}_{2}+{H}_{2}{O}_{2}$$

8 $${H}_{2}{O}_{2}+{O}_{2}^{\cdot -}\to O{H}^{\cdot }+O{H}^{-}+{O}_{2}$$

9 $${H}_{2}{O}_{2}+Ti{O}_{2}({e}_{CB}^{-})\to Ti{O}_{2}+O{H}^{\cdot }+O{H}^{-}$$

10 $${H}_{2}O+Ti{O}_{2}({h}_{vB}^{+})\to Ti{O}_{2}+O{H}^{\cdot }+{H}^{+}$$

11 $$O{H}^{-}+Ti{O}_{2}({h}_{vB}^{+})\to Ti{O}_{2}+O{{\rm{H}}}^{\cdot }$$

12 $${\rm{Dye}}+O{{\rm{H}}}^{\cdot }\to degradation\,product$$

13 $${\rm{Dye}}+Ti{O}_{2}({h}_{vB}^{+})\to oxidation\,production$$

**Figure 7:**
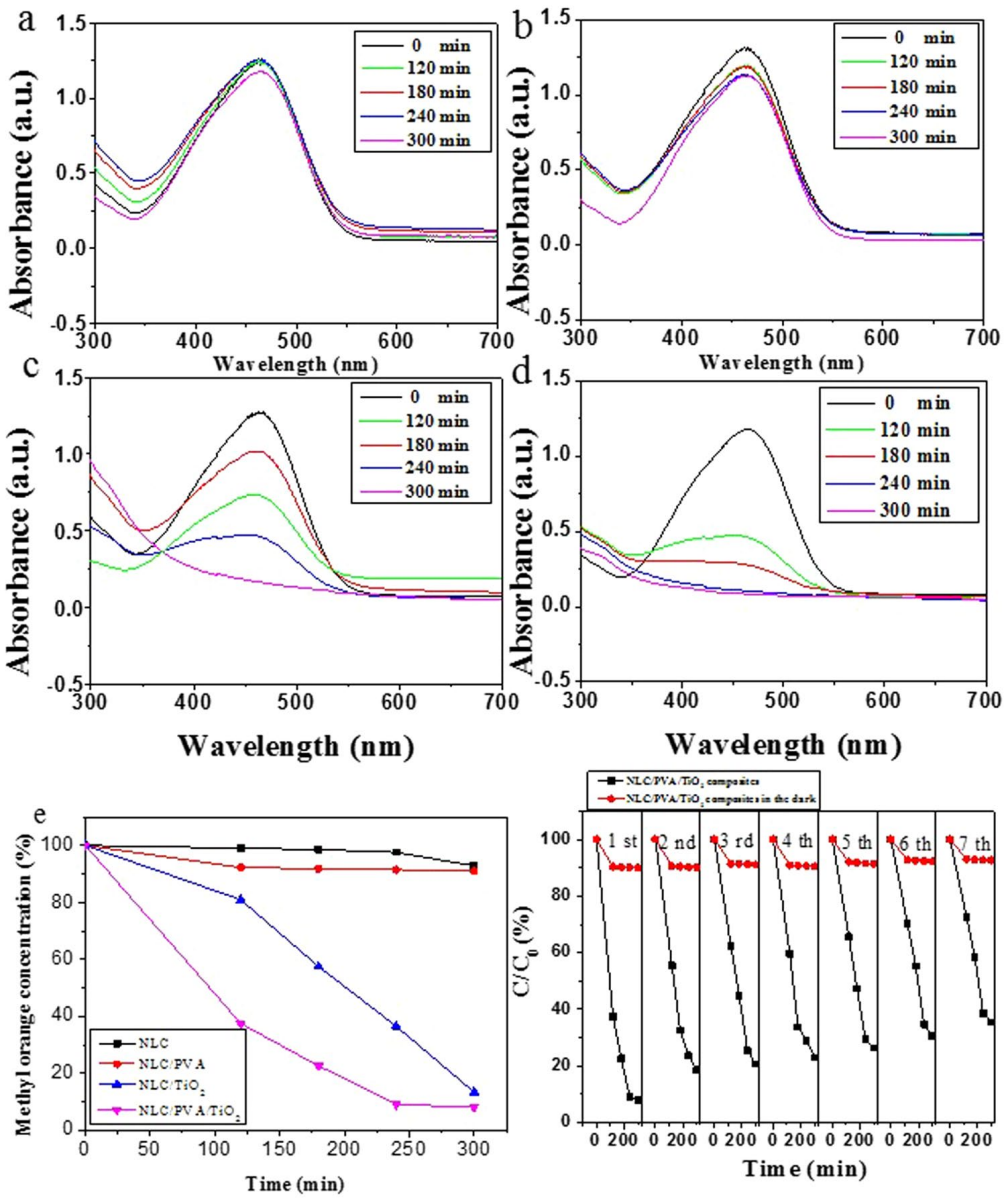
(**a**–**d**) UV–visible spectra of the control (methyl orange) and its degradation by NLC, NLC/PVA composite, NLC/TiO_2_ composite and NLC/PVA/TiO_2_ composite, respectively. (**e**) Quantitative study of photocatalytic degradation of methyl orange by NLC, NLC/PVA composite, NLC/TiO_2_ composite and NLC/PVA/TiO_2_ composite as a function of UV irradiation time. (**f**) Seven cycles of the photodegradation of MO using NLC/PVA/TiO_2_ as photocatalyst under UV irradiation for 300 min.

In order to probe the effectiveness of these composites for this photocatalytic application, the composites (20 mm × 7 mm × 5 mm) strips were dipped in an aqueous solution of MO (0.15 mM) and then exposed to the was exposed to the UV irradiation produced by a tunable pressure Hg lamp, at 1000 w. In Fig. 7a and b the NLC and NLC/PVA composites showed almost no degradation of methyl orange. As for the NLC/TiO_2_ (Fig. 7c) and NLC/PVA/TiO_2_ (Fig. 7d) composites, it was observed that the intensity of λ_max_ decreases on increasing the exposure time. Moreover, this excluded the influence of matters in NLC and PVA on photocatalytic activity. The photodegradation curves of methyl orange by NLC, NLC/PVA, NLC/TiO_2_ and NLC/PVA/TiO_2_ composites under UV light irradiation are shown in Fig. 7e, NLC/TiO_2_ and NLC/PVA/TiO_2_ composites degraded approximately 86.6% and 92% of methyl orange, respectively, under the UV irradiation time of 300 min. The methyl orange degradation was due to the shortlived methyl orange cation, which spontaneously decomposes on injecting an electron into the conduction band of TiO_2_. The results showed the degradation efficiency of methyl orange of NLC/PVA/TiO_2_ composites.

The stability and reusability of the NLC/PVA/TiO_2_ composites were measured by monitoring the degradation of methyl orange. In each cycle, simulated UV light was irradiated for 300 min at room temperature in Fig. 7f. After irradiation for 5 h, the MO was degraded up to 91.9% by NLC/PVA/TiO_2_composites, after 7 cycles, the catalyst maintains photoactivity (64.6%), these results demonstrated excellent stability and reusability of NLC/PVA/TiO_2_ composites. In addition, there was no obvious photodegradation by NLC/PVA/TiO_2_ composites as-prepared in darkness (red lines in Fig. 7f), and NLC/PVA/TiO_2_ composites had a gradually diminishing adsorption capacity for MO when placed in the dark after 7 cycles.

The UV-vis DRS of the hybrid were shown in Figure S2. The nanolignocellulose/PVA/TiO_2_ composites showed absorption peak at 400 nm ultraviolet band, which confirm its photo-absorption performance. The LC chromatogram of the degraded methyl orange and mass spectra and compound confirmation for the degradation products were shown in Figure S3. The results were further supported by LC-MS. The degraded products were separated at the retention times of 5.8 min and 7.9 min, each peak was characterized using its mass measurements (m/z) (Figure S3). In Figure S3b, it was concluded that the degradation pathway of methyl orange firstly involved in a symmetric cleavage of the azo bond yielding benzenesulfonic acid at m/z 157 (C_6_H_5_SO_3_). The other degradation product was N, N-dimethyl benzenamine, which was confirmed via a mass measurement at m/z 121 (C_8_H_11_N) with a characteristic fragment at m/z 106 (Figure S3c). Therefore, the results direct evidenced for the degradation ability of the composite.

## Discussion

In conclusion, inspired by the layered aragonite platelet/nanofibrillar chitin/protein structure of nacre, nacre-like composites based on PVA/NLC/TiO_2_ through hot-pressing process were constructed. PVA and TiO_2_ nanoparticles have been used as nanofillers to improve the mechanical of composites and endow photocatalytic performance to composites. The nacre-like composites obtained have a nacre-like bricks-and-mortar microstructure, which leads to achieve an excellent mechanical property. The binding strength, elasticity modulus and internal bond strength reach 24.6 ± 5 MPa, 1255.5 ± 41 MPa and 0.87 ± 0.04 MPa, respectively, which surpass other layered cellulose/polymer binary composites. On the other hand, the photocatalytic properties make these composites promising candidates in the photooxidation of volatile organic compounds (VOCs) and other organic pollutants applications.

## Methods

### Materials

Lignocellulose based on softwood was obtained in a dried form, poly (vinyl alcohol) was supplied by Sinopharm Chemical Reagent Co., Ltd, titania (TiO_2_) and methyl orange were purchased from Aladdin (Shanghai, China).

### Fabrication of nacre-like composites

The lignocellulose suspension mixed with 1 wt.% TiO_2_ and 4 wt.% PVA was added into SuperMassColloider at 1,500 rpm. The SuperMassCollider was equipped with a power meter to record electrical energy input^27^. Two metallic grinding disks were positioned to concentric circles. The inboard disk rotated while the outboard one was stationary. Suspension feeding was achieved by gravity. A hydraulic pressure head could also be developed for runs with large capacity when the flow loop was properly constructed. The gap of the two disks was adjusted to −100 μm from motion zero position after suspension was loaded. The motion zero position was determined right at the contact position between the two grinding disks before loading the suspension. Due to the presence of suspension, there was no direct contact between the two grinding stones even at a negative setting of disk position^27^. Lignocellulose/PVA/TiO_2_ suspension was fed into the disk grinder continuously for 6 hours through a loop consisting of a peristaltic pump and plastic tubing. Finally, after the redundant water of NLC/PVA/TiO_2_ suspension was filtered, the composites were hot-pressed at 200 °C, 2.5 MPa and cured into the layered board.

### Fracture testing

The nacre-like composites was measured 10 mm × 60 mm × 5 mm, the fracture tests were performed using a three-point bending fixture mounted on a miniature loading stage (Reger RGM-6010T) with a span length of 30 mm, and the specimens were loaded at a rate of 0.05 mm s^−1^ up to failure. For each composition, more than 10 samples were tested, from which the mean and standard deviation were calculated.

### Determination of internal bond (IB) strength

IB tests were conducted on specimens cut from the nacre-like composite. Loading blocks of stell 25 mm square and 5 mm in thickness were effectively bonded with polyvinyl acetates glue to the 25 mm square faces of the specimens. Mechanical tests were carried out on a Losenhausen Universal testing system equipped a load cell with a capacity of 1000 kg, manufactured by CRIMS CH DNS50. The load was continuously applied to the specimens throughout the tests at a uniform rate of motion of the movable cross-head of the testing machine of 1.2 mm/min until failure occurs. IB strength of the specimen was determined based on the following equation^28^:

14 $${f}_{v}=P/A$$

Where *f*_v_ is the IB strength (N/mm^2^), P the load at which the specimen failed (N), and the surface area of the specimen (mm^2^).

### Photocatalytic activity

The photocatalytic activity of the NLC/PVA/TiO_2_ composite against methyl orange was carried out under UV light illumination. Typically, the composite were cut to 20 mm × 7 mm × 5 mm dimensions and were dipped in the aqueous solution of methyl orange (0.25 mM in 4 mL water). The solution was then exposed to a 50 cm long 72 W UV-A lamp with an emission range of 320–400 nm from the distance of 30 cm for the time duration of 120–300 min. Methyl orange does not absorb UV light; however, TiO_2_ absorbs between 350 and 400 nm^29^. The degradation of methyl orange (λ_max_ at 464 nm) was studied with a UV-Vis spectrophotometer (Pgeneral TU-1901 UV-Vis spectrophotometer) in the range of 200–800 nm.

### Characterizations

The surface morphology of the composites was studied using Scanning Electron Microscopy (SEM, FEI, Quanta 200, USA). X-ray diffraction (XRD)patterns were measured on a Bruker D8 Advance with Cu-Kα radiation (λ = 1.5409 Å) diffraction meter. The high-resolution transmission electron microscopy (HRTEM) images from the Tecnai G2 F20 were used to obtain crystallographic information. X-ray photoelectron spectroscopy (XPS) was carried out on a ThermoFisher K-Alphato characterize the valence state of elements and depth compositional profiles of films. The three-point bending tests were evaluated using aRegerRGM-6010T, internal bond strength tests were evaluated using a CRIMS CHDNS50.

## Electronic supplementary material

Supporting Imformation of Bio-Inspired nacre-like nanolignocellulose-poly (vinyl alcohol)-TiO2 composite with superior mechanical and photocatalytic properties
