# Supplementary material for: Bio-Inspired nacre-like nanolignocellulose-poly (vinyl alcohol)-TiO2 composite with superior mechanical and photocatalytic properties
Source: Sci Rep. 2017 May 12;7:1823. doi: 10.1038/s41598-017-02082-8 (PMC5431784; doi:10.1038/s41598-017-02082-8)
Supplement: Supplementary file 1 — Supporting Imformation of Bio-Inspired nacre-like nanolignocellulose-poly (vinyl alcohol)-TiO2 composite with superior mechanical and photocatalytic properties [file 41598_2017_2082_MOESM1_ESM.doc]

Correspondence and requests for materials should be addressed to: Q.F. Sun ([qfsun@zafu.edu.cn](mailto:qfsun@zafu.edu.cn)); C.D. Jin (zafujincd@163.com)

**Title: Bio-Inspired** **nacre-like** **nano****lignocellulose-poly (vinyl alcohol)-TiO_2_** **composite with superior mechanical and photocatalytic properties**

Author names: Yipeng Chen^1^, Hanwei Wang^1^, Baokang Dang^1^, Ye Xiong^1^, Qiufang Yao^1^, Chao Wang^1^, Qingfeng Sun^1,2*^, Chunde Jin^1,2*^

Affiliations: 1. School of Engineering, Zhejiang A & F University, Hangzhou, Zhejiang Province, 311300, PR China; 2. Key Laboratory of Wood Science and Technology, Zhejiang Province, 311300, PR China

Figure S1 the FTIR spectra of the NLC, NLC/PVA, NLC/TiO_2_ and NLC/PVA/TiO_2_ composites

Figure S2 UV-vis DRS of nanolignocellulose/PVA/TiO_2_ composites


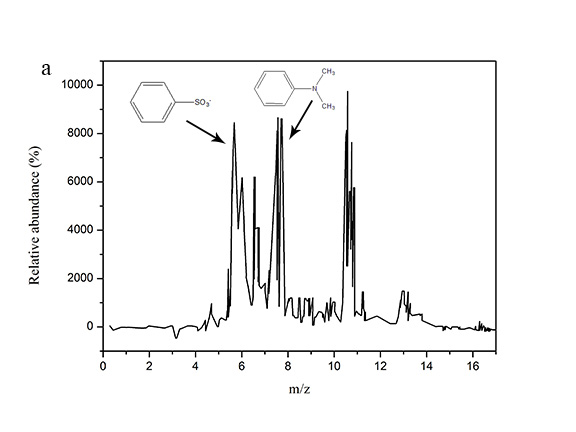


Fig. S3 (a) LC chromatogram of the degraded methyl orange, (b and c) the mass spectra and compound confirmation for the degradation products
